# Supplementary material for: Causal Relationship between Aging and Anorexia Nervosa: A White-Matter-Microstructure-Mediated Mendelian Randomization Analysis
Source: Biomedicines. 2024 Aug 16;12(8):1874. doi: 10.3390/biomedicines12081874 (PMC11351342; doi:10.3390/biomedicines12081874)
Supplement: Supplementary file 1 [file biomedicines-12-01874-s001.zip › Supplementary Figure/Figure S4/Singe SNP plot of MR analysis for TL to ubm-b-1507.pdf]

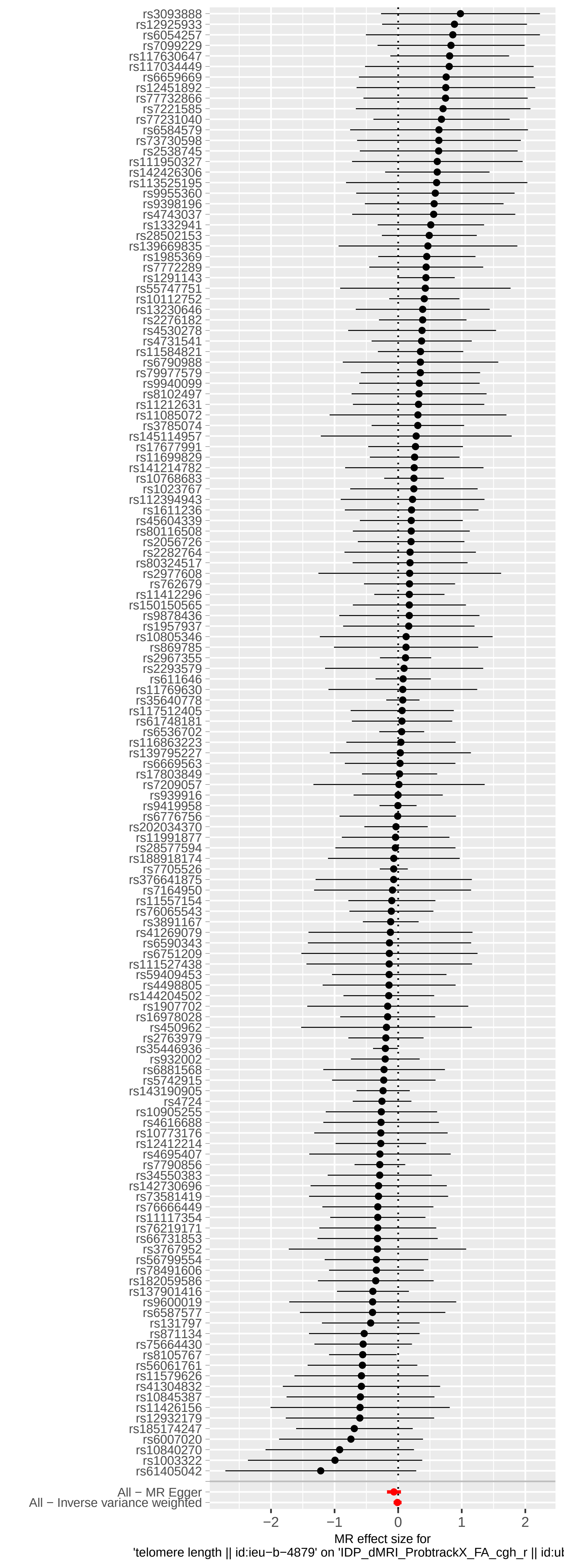
rs3093888
rs12925933
rs6054257
rs7099229
rs117630647
rs117034449
rs6659669
rs12451892
rs77732866
rs7221585
rs77231040
rs6584579
rs73730598
rs2538745
rs111950327
rs142426306
rs113525195
rs9955360
rs9398196
rs4743037
rs1332941
rs28502153
rs139669835
rs1985369
rs7772289
rs1291143
rs55747751
rs10112752
rs13230646
rs2276182
rs4530278
rs4731541
rs11584821
rs6790988
rs79977579
rs9940099
rs8102497
rs11212631
rs11085072
rs3785074
rs145114957
rs17677991
rs11699829
rs141214782
rs10768683
rs1023767
rs112394943
rs1611236
rs45604339
rs80116508
rs2056726
rs2282764
rs80324517
rs2977608
rs762679
rs11412296
rs150150565
rs9878436
rs1957937
rs10805346
rs869785
rs2967355
rs2293579
rs611646
rs11769630
rs35640778
rs117512405
rs61748181
rs6536702
rs116863223
rs139795227
rs6669563
rs17803849
rs7209057
rs939916
rs9419958
rs6776756
rs202034370
rs11991877
rs28577594
rs188918174
rs7705526
rs376641875
rs7164950
rs11557154
rs76065543
rs3891167
rs41269079
rs6590343
rs6751209
rs111527438
rs59409453
rs4498805
rs144204502
rs1907702
rs16978028
rs450962
rs2763979
rs35446936
rs932002
rs6881568
rs5742915
rs143190905
rs4724
rs10905255
rs4616688
rs10773176
rs12412214
rs4695407
rs7790856
rs34550383
rs142730696
rs73581419
rs76666449
rs11117354
rs76219171
rs66731853
rs3767952
rs56799554
rs78491606
rs182059586
rs137901416
rs9600019
rs6587577
rs131797
rs871134
rs75664430
rs8105767
rs56061761
rs11579626
rs41304832
rs10845387
rs11426156
rs12932179
rs185174247
rs6007020
rs10840270
rs1003322
rs61405042
All − MR Egger
All − Inverse variance weighted
−2
−1
0
1
2
MR effect size for
'telomere length || id:ieu−b−4879' on 'IDP_dMRI_ProbtrackX_FA_cgh_r || id:u
